# The Wedding Trip

**Published:** 1887-05

**Authors:** 


### *THE WEDDING TRIP.*

The French medical journals and some of the English have been lately calling attention to the evils of the wedding trip.

There are few physicians who will not recall many cases in which a girl, perfectly healthy till her marriage and a long wedding trip, is never healthy again. The number of women who date a life of chronic invalidism to a wedding trip is not small. So apparent have been these evils, that it is reported a custom has arisen by which the demands of fashion for a wedding trip shall be complied with, and yet the newly-married couple enjoy a period of repose and quiet all by themselves. The plan is to make ostensible arrangements for a trip, and even drive to the station, but in reality turn back to a hotel or some intimate friend's, in which, all alone by themselves, the newly-married couple shall begin their life journey.

Marriage is one of the epochs of life. It is peculiarly related to the physical well-being of both parties and to the unborn. To the young wife, there has been long and exhausting excitement in arranging for the event. To this is added an entrance upon physical relations utterly new to her. Surely this is quite enough to bear in the retirement of a quiet home, or away from inquiring acquaintances. Surely this is enough without the discomfort of railway travel, the exhaustion of hurrying from place to place, the excitement of new scenes and people, and the exposure to extremes of heat or cold, of storms, and all sorts of annoyances inseparable from long journeys.

We have often thought that physicians, by giving a word of friendly advice to such of their patients as chanced to be about to enter upon a married life, might be the means of saving such persons from future misery. Family physicians are the ones to reach these cases. True, they would have to combat social customs, but after all we think that in the end they would win.—*American Lancet.*

